# The rheumatoid arthritis citrullinome is enriched in antigenic complement proteins

**DOI:** 10.1186/s13075-026-03750-9

**Published:** 2026-01-28

**Authors:** Mario Navarrete, Khushali Trivedi, Clarissa Klenke, Zihao Zheng, Jun Kim, Jeba Atkia Maisha, Alina Semenenko, Xiaobo Meng, Obinna I. Okeke, Carolina Munoz-Grajales, Christine Peschken, Miriam A. Shelef, Hani S. El-Gabalawy, Liam J. O’Neil

**Affiliations:** 1 Department of Internal Medicine, University of Manitoba, RR-149 800 Sherbrook St. R3E1R9, Winnipeg, Canada; 2 Department of Medicine, University of Wisconsin-Madison, Madison, WI USA; 3 Department of Statistics, University of Wisconsin-Madison, Madison, WI USA; 4 Department of Immunology, University of Manitoba, Winnipeg, Canada; 5 William S. Middleton Memorial Veterans Hospital, Madison, WI USA

## Abstract

**Objectives:**

Citrullination is a post-translational modification that serves as an autoantigen in Rheumatoid Arthritis (RA). It remains unclear whether there is an excess production of specific citrullinated proteins in RA patients that is driving the autoantibody response. The aim of this study was to undertake an unsupervised proteomic analysis of the circulating RA citrullinome, and determine the consequences of citrullination in RA.

**Methods:**

Citrullinated serum proteins were isolated and measured by mass spectrometry from 10 anti-citrullinated protein antibody (ACPA) positive RA patients and 10 controls. Cit-proteins and cit-immune complexes (IC) were measured in a larger cohort of unaffected first-degree relatives (FDR, ACPA- *n* = 31, ACPA+ *n* = 26) and RA (*n* = 31). Autoantibodies to citrullinated C9, and linear peptides of citrullinated C9 were measured. Structural changes imparted by citrullination were studied in-silico using Alphafold 3.0.

**Results:**

Differential analysis of the RA citrullinome revealed a higher expression of complement proteins. Validation studies revealed that the levels of cit-C9 (*p* = 0.02) and cit-CFI (Complement Factor I, *p* = 0.019) were higher in RA than controls. Cit-C9 IgG, but not cit-CFI, immune complexes were elevated in ACPA+ FDR (*p* < 0.0001) and RA (*p* < 0.0001) compared to ACPA- FDR. Autoantibodies to cit-C9 were also higher in ACPA+ FDR (*p* = 0.01) and RA (*p* = 0.002). Reactivity to linear peptides of cit/homocit C9 were enriched in RA patients compared to controls. In-silico modelling revealed structural changes to the monomeric and multimeric structure of cit-C9.

**Conclusion:**

This analysis of the circulating citrullinome in RA reveals the generation of citrullinated complement proteins that serve as autoantigens.

**Supplementary Information:**

The online version contains supplementary material available at 10.1186/s13075-026-03750-9.

## Introduction

Rheumatoid Arthritis (RA) is a chronic, systemic autoimmune disease that leads to progressive inflammatory polyarthritis [1]. Most patients with RA exhibit autoantibodies that are reactive to proteins containing citrulline, an irreversible post-translational modification of arginine [2]. These autoantibodies are termed anti-citrullinated protein antibodies (ACPA). ACPA are the hallmark of seropositive RA and are associated with more severe and difficult to control disease, while also serving as an important biomarker for diagnosing the disease at the onset of clinical arthritis [3]. It is now well known that in individuals with seropositive RA, ACPAs develop during the pre-clinical stage of RA, in some cases years before the onset of clinical arthritis [4].

Despite the well-established relationship between protein citrullination and autoantibody responses in RA, the role of citrullination itself in RA pathogenesis remains incompletely understood. Citrullination is a ubiquitous biological processcommon biological event, and this post-translational modification plays a key role in RNA transcription [5], histone modification for epigenetic regulation [6], immune regulation [7], along with other functions such as cornification of the skin [8]. Despite these ubiquitous physiological functions, the generation of specific citrullinated epitopes in vivo may, in predisposed individuals, lead to the development of RA autoimmunity and its inflammatory consequences. Thus, the delineation of alterations in circulating citrullinated proteins (*citrullinome*) that are exhibited by seropositive RA patients may provide novel insights into how and why this physiological process may potentially become pathogenic in affected individuals.

The purpose of the current study is to characterize the serum citrullinome of RA patients and compare it to the serum citrullinome of unaffected controls. Using a purification process that involves a citrulline-specific probe, we identified by mass spectrometry an overabundance of proteins that included complement proteins. Our findings further suggest that citrullination of complement factor 9 alters the antigenicity of the protein in seropositive RA patients and in ACPA+ unaffected individuals. Moreover, in-silico analysis suggests that citrullination impacts protein folding of monomeric and multimeric C9 structures (membrane attack complex or MAC).

## Methods

### Experimental design and statistical rationale

#### Cohorts

Serum samples used for this study were derived from a well-established longitudinal study of RA risk [4, 9]. In brief, RA patients (probands) were approached to recruit their First-Degree Relatives (FDR) who were then followed prospectively. Due to shared genetics and environmental risk factors, FDR are presumed to have a higher-than-average risk to develop RA. A proportion of these individuals were ACPA positive at baseline or developed ACPA during longitudinal follow up (ACPA+ FDR), which we have previously shown is a dominant risk factor for RA development [4]. Indeed, a small number of individuals did develop RA during the follow up period, which has been reported in prior publications [4]. To profile the serum citrullinome by mass spectrometry, samples were selected from 10 patients with RA who were matched by age and sex to 10 FDR controls (Table 1). This corresponds to detecting proteins with approximately a 2-fold difference in abundance, assuming moderate variability (coefficient of variation ≈ 25–30%). The median age was 42.4 years old, and the majority of patients were female (80%). Validation studies were conducted on a separate subset of 88 individuals, 32 ACPA- FDR, 25 ACPA+ FDR and 31 RA probands from the same cohort. A previously published peptide array dataset was used to validate RA antibody responses to cit-C9, which included 48 RA patients and 12 controls, details of this cohort can be find in the past publication [10]. All samples were collected in a protocol approved by the Research Ethics Board at the University of Manitoba (Canada).

**Table 1:** Baseline demographics for serum samples

	Mass Spectrometry		Validation Cohort		
	ACPA- FDR (*n* = 10)	RA Proband (*n* = 10)	ACPA- FDR (*n* = 32)	ACPA+ FDR (*n* = 25)	RA Proband (*n* = 31)
Age, median (IQR)	41.8 (14.0)	43.0 (14.0)	42.6 (14.4)	42.04 (13.8)	46.4 (14.3)
Sex, F	8 (80%)	8 (80%)	20 (62.5%)	23 (92.0%)	24 (77.4%)
ACPA positive	0 (0%)	10 (100%)	-	25 (100%)	21 (67.8%)
TJC28, median (IQR)	-	7.5 (8.3)	2 (10.5)	2 (8)	8 (14.3)
SJC28, median (IQR)	-	3 (5.5)	-	-	2 (8)
DAS28, median (IQR)	-	4.2 (1.1)	-	-	4.2 (2.3)

### Identification of citrullinated serum proteins by mass spectrometry

Serum proteins were quantified via bicinchoninic acid (BCA, Pierce), passed through a 0.22 μM filter and treated with a protein depletion kit which removes the top 14 most abundant proteins (High-Select) following the manufacturer’s instructions. These abundant proteins, while essential for overall plasma homeostasis including immunoglobulins, fibrinogen, albumin, haptoglobin and others, are not typically implicated in citrullination pathways relevant to autoimmune disease pathogenesis. Depletion was performed to improve mass spectrometry sensitivity for detecting lower-abundance citrullinated proteins. 300 ug of depleted sera were labelled using a biotin-phenylglyoxal probe (5 mM, Cayman) with 100% Trichloroacetic acid at a protein concentration of 1μg/μL. Following cold acetone washes, protein pellets were resuspended in 1.2% Sodium dodecyl sulfate and boiled for 10 min followed by sonication for 15 min. Samples were then diluted in phosphate buffered saline (PBS, pH 7.4) and incubated with 170 μL of streptavidin agarose slurry overnight at 4°C. The beads were then washed with PBS (0.2% SDS), 8M urea, and reduced with 100 mM Dithiothreitol at 60°C for 30 min. Samples were alkylated with 500 mM Iodoacetamide for 45 min at room temperature. On bead digestion was undertaken with trypsin at 37°C overnight, washed subsequently with digestion buffer (50 mM Tris), 50% acetonitrile and 1% Trifluoroacetic acid solution with all fractions added together. Samples were then lyophilized and stored at -80°C until MS analysis. See supplemental methods for mass spectrometry details.

Raw files were uploaded to MaxQuant for processing using the following settings: minimum peptide length (7 amino acids), maximum peptide mass (4600 Da), label free quantification (LFQ) mininum ratio 2. Modifications included carbamidomethyl (C), Acetyl (Protein N-term), Carbamyl (N-term), Deamidation (NQ), Oxidation (M) and Dioxidation (MW). Match between runs was enabled. LFQ data was then processed in R using the *DEP* package. The matrix of proteins was filtered based on expression values within each group (RA and controls), and missing data was imputed using k-nearest neighbour.

### Proteomic data analysis

To visualize patterns of similarity among samples, we applied classical multidimensional scaling (MDS) to the dataset. The protein features were extracted and converted to a distance matrix using Euclidean distance. MDS was then performed via the cmdscale() function in R which were plotted to assess potential clustering of samples based on overall similarity. Unsupervised consensus clustering was performed using the ConsensusClusterPlus R package. We employed k-means clustering (Euclidean distance) with up to 8 potential clusters (maxK = 8), using 50 resampling iterations, subsampling 80% of samples per iteration (pItem = 0.8). The optimal number of clusters was selected based on inspection of consensus matrices and cumulative distribution functions. Cluster assignments were integrated with the MDS coordinates to enable visualization of clustering structure within the reduced dimensional space.

To identify groups of co-expressed or strongly correlated features, we employed a correlation-based feature selection approach. A pairwise correlation matrix was generated using Pearson’s correlation for all features. The matrix was then converted into a graph structure, with variables represented as nodes and edges drawn between nodes if their absolute correlation exceeded a predefined threshold (|r| > 0.7). Maximal cliques (fully connected subgraphs) of size 8 to 20 were identified using the igraph package in R. This approach allowed us to detect clusters of variables that exhibit strong inter-correlations, potentially reflecting shared biological or functional relationships. From these cliques, a specific subset of highly correlated variables was selected for further analysis and visualization. These variables were visualized using heatmaps of the corresponding correlation matrix to highlight intra-clique relationships.

### In-silico modelling of C9 and citrullinated C9

To determine the impact of protein citrullination on the tertiary structure of C9 (Uniprot ID: P02748) we used Alphafold 3.0 (alpha fold server) to model native and citrullinated C9. Citrulline was introduced using the PTM function in alpha fold and .cif files were downloaded and converted to .pdb files. PDB files were viewed and analyzed in Research Collaboratory for Structural Bioinformatics Protein Data Bank Molecular 3D viewer (https://www.rcsb.org/3d-view). A similar process was undertaken to model the membrane attack complex, which is a pore formed by several subunits of C9. PDB files were also analyzed in R using bio3d. Structures were aligned using *fit.xyz()* and global root-mean square deviation (RMSD) per residue were calculated from aligned coordinates. B-factors were extracted directly and compared between structures. Torsion angles were computed using *torsion.pdb()* and secondary structure was inferred from angle-based rules to quantify residue-level helix, sheet and coil differences, and visualized with ggplot2.

### Other statistical analysis

Group level statistics were calculated using student t-test for parametric data and Wilcoxon-rank sum test for non-parametric data. Spearman was used to correlate continuous variables. Hierarchical clustering, dimensionality reduction, differential expression (pairwise Wilcoxon rank-sum test) was undertaken using R (RStudio). See supplementary files for extended methods.

## Results

### The RA citrullinome is enriched with complement proteins

**Fig. 1:**
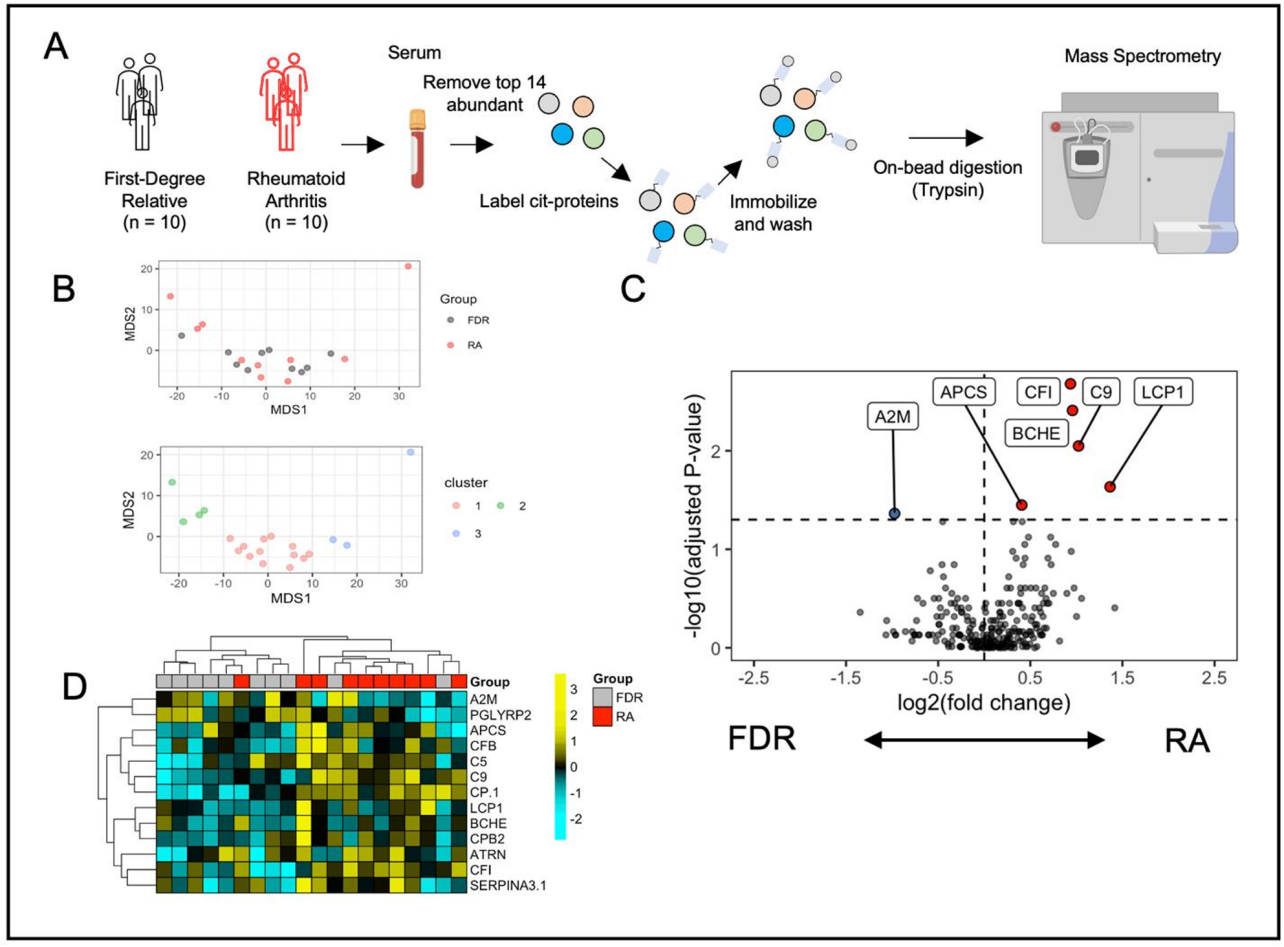
Evaluation of the Rheumatoid Arthritis citrullinome. (**A**) Experimental outline of sera used for isolation of citrullinated proteins and mass spectrometry for peptide identification. (**B**) Multi-dimensional scaling of FDR controls and RA samples (upper) and clusters defined by hierarchical clustering (lower). (**C**) Volcano plot of differentially expressed citrullinated proteins in RA compared to FDR. (**D**) Heat map and hierarchical clustering of RA and control serum samples based on levels of serum citrullinated proteins

To investigate the circulating human citrullinome, we enriched citrullinated proteins from a total of 20 individuals: 10 individuals with established, seropositive RA, and 10 unaffected First-Degree Relatives (FDR) of RA patients with no evidence of arthritis or detectable RA-associated autoantibodies, who were being followed in the context of a longitudinal study of RA onset [4]. Proteins were labelled, immobilized on streptavidin beads and processed for mass spectrometry (Fig. 1A). A total of 250 proteins were identified in these 20 individuals. Principle components analysis using multi-dimensional scaling revealed 3 distinct clusters of individuals, with cluster 2 and 3 combined being over-represented with the RA patients (5/7, 71.4%, Fig. 1B). Using the entire cohort, we used correlation-based feature selection to identify protein networks in the citrullinome, and identified three networks arbitrarily referred to as A, B and C (Fig. S1), with Network B enriched in *complement activation* (*p* = 2.5 × 10^− 7^) and Network C enriched in *protein processing* (*p* = 0.007, Fig. S2). We observed a higher intra-network correlation in RA samples within Network A (*p* = 0.001) and FDR within Network B (*p* < 0.0001), suggesting differences in protein-protein networks depending on disease state (Fig. S3).

**Fig. 2:**
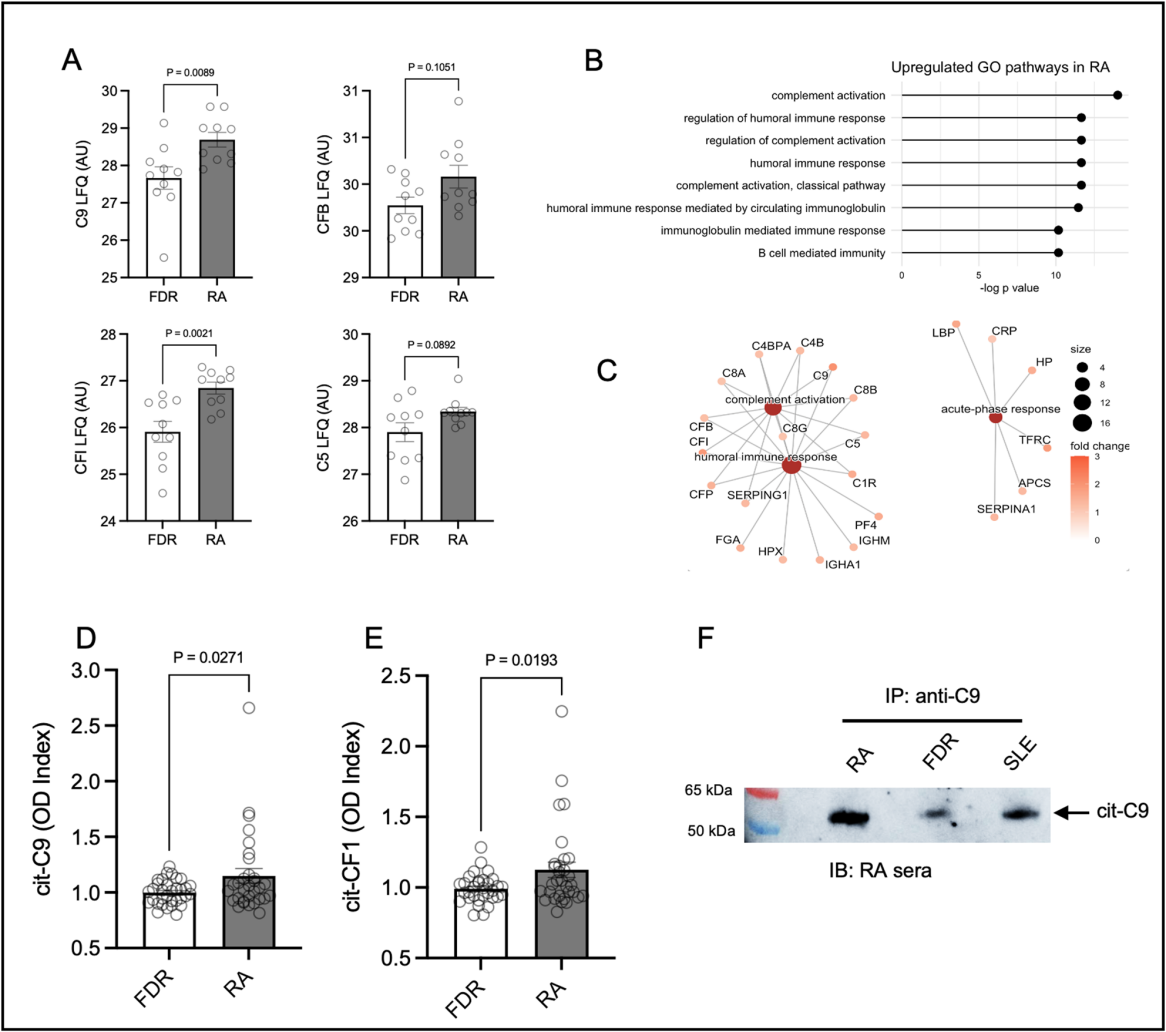
The Rheumatoid Arthritis citrullinome is enriched with citrullinated complement proteins. (**A**) Levels of citrullinated proteins in RA compared to FDR controls as measured by label-free quantification (LFQ) for C9, CFB, CFI and C5. (**B**) Gene ontology (GO) pathways enriched in the citrullinome of RA patients and (**C**) protein-protein interaction plots of select GO pathways. (**D**) Confirmatory ELISA testing of citrullinated C9 levels and (**E**) citrullinated CFI levels in serum of RA patients and controls. (**F**) Western blot of immunoprecipitated C9 from pooled sera of RA, FDR and SLE patients, immunblotted with RA sera from an ACPA+ patient

We next sought to investigate individual citrullinated proteins that are enriched in RA sera. Differential expression analysis identified 6 proteins (2.4% of the total citrullinome) which were differentially expressed in RA versus controls, 5 of which were increased in RA and 1 of which was decreased (Fig. 1C). Hierarchical clustering of samples based on differentially expressed cit-proteins identified a cluster enriched in RA (Fig. 1D and 9/11 81.2%, Cluster 2). Notably, the proteins increased in RA included complement factor I (CFI, Fig. 2A) and complement 9 (C9), along with serum amyloid P (APCS), Butrylcholinesterase and lymphocyte cytosolic protein (LCP1). Alpha-2-macroglobulin, a protease inhibitor, was decreased in RA. Given that 2 of the differentially expressed proteins were related to the complement cascade, we profiled the presence of other complement proteins that trended towards enrichment in RA, finding that citrullinated Complement factor 5 (C5) and Complement factor B trended higher in RA sera compared to controls (Fig. 2A, *p* = 0.09, *p* = 0.1 respectively).

We next performed a network analysis on upregulated gene ontology pathways in the RA citrullinome. 92 upregulated pathways were identified which included *complement activation* (Fig. 2C, *p* = 2.1 × 10^− 20^), *humoral immune response* (*p* = 3.8 × 10^− 20^), and the *acute phase response* (*p* = 2.7 × 10^− 9^, Table S1) Protein interaction networks revealed linkage between citrullinated proteins involved in the complement cascade and the humoral immune response suggestive of potential protein-protein interactions that mediate host defense (Fig. 2C), however the acute-phase response proteins, including CRP, while also increased, were found to be a distinct network.

To verify the abundance of the 2 highest complement proteins (C9 and CFI) in our mass spectrometry data, we used a plate-based ELISA to sandwich total citrullinated proteins and either C9 or CFI (see Methods) using a validation cohort of RA and controls (both ACPA negative and ACPA positive). We observed an increase in both citrullinated C9 and citrullinated CFI (Fig. 2D and E) in RA when compared to controls (*p* = 0.03 and *p* = 0.02 respectively). We did not observe a significant correlation between DAS28 scores and cit-C9 or cit-CFI levels (Fig. S4), but we did find a strong correlation between the levels of cit-C9 and cit-CFI in the RA cohort (Fig. S4, *R* = 0.69, *p* < 0.0001). Given the importance of citrullination and autoreactivity to citrullinated proteins/peptides in preclinical RA [4], we also sought to determine if cit-C9 and cit-CFI were also increased in ACPA+ controls without RA. However, compared to ACPA- controls, we found no differences in the levels of either citrullinated complement protein in ACPA+ FDR (Fig. S5). To further verify that citrullinated C9 is present in RA sera, we immunoprecipitated total C9 from pools (*n* = 3) of serum from RA, FDR and SLE, a B-cell mediated autoimmune disease associated with complement activation [11]. A western blot using serum IgG from an ACPA+ RA patient revealed high levels of citrullinated C9 in RA compared to FDR, with an intermediate profile for SLE (Fig. 2F). Overall, these data suggest that citrullinated complement proteins are enriched in RA patients but do not correlate with disease activity.

### Citrullinated complement proteins in RA form immune complexes with terminal product C9

**Fig. 3:**
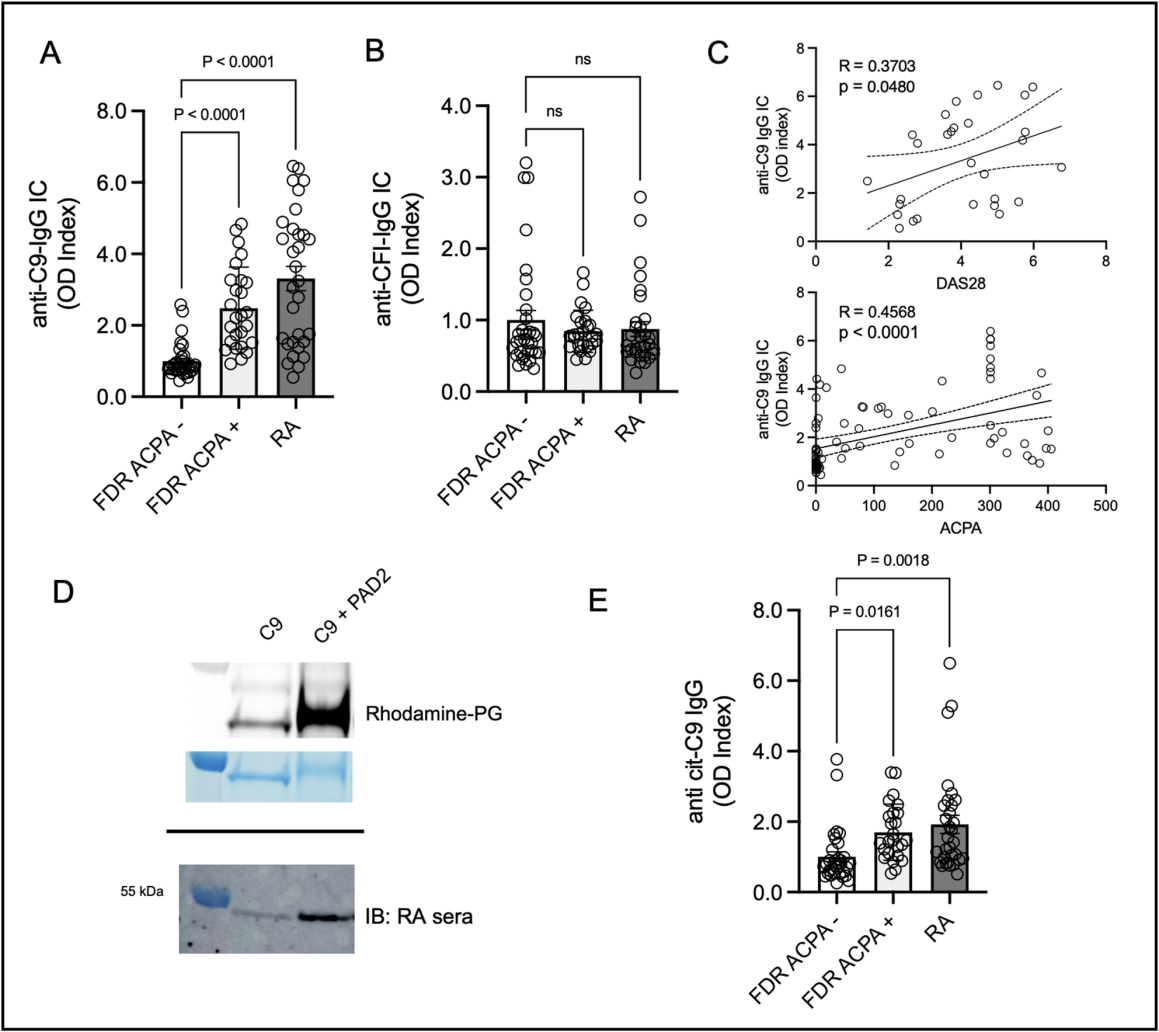
Citrullinated C9 is an autoantigen in RA and ACPA+ controls without RA. (**A**) IgG Immune complexes of C9 are increased in the serum of RA and ACPA+ controls, but not ACPA- controls. (**B**) IgG immune complexes of CFI are not increased in RA and ACPA+ controls compared to ACPA- controls. (**C**) IgG immune complexes of C9 correlate with DAS28 scores (upper) in established RA and correlate with ACPA levels in the serum of RA patients and ACPA+ controls without RA (lower). (**D**) Confirmation of citrullination of C9 using Rhodamine-Phenylglyoxal probe in-gel with and without treatment with PAD2 (upper) and reactivity to RA sera from an ACPA+ individual by western blot. (**E**) Levels of autoantibodies citrullinated C9 in ACPA- controls, ACPA+ controls and RA patients measured by ELISA

Given the importance of IC formation and complement activation in RA pathogenesis [12] we next sought to determine if immune complexes (IC) that target citrullinated C9 or CFI are detectable in RA sera. First, we aimed to determine if immunocomplexes (IC) bound to native complement C9 or CFI were elevated in RA. We observed a significant increase in anti-C9 IC’s (IgG) in RA and ACPA+ controls compared to ACPA- controls (Fig. 3A, *p* < 0.0001 for both comparisons) but did not observe any differences in anti-CFI ICs in either group (Fig. 3B). In the RA cohort, we found that anti-C9 ICs correlated positively with DAS28 scores, a key measure of RA disease activity (Fig. 3C, *R* = 0.37, *p* = 0.048). We also observed a positive correlation between anti-C9 ICs and ACPA levels (Fig. 3C, *R* = 0.46, *p* < 0.0001). We thus hypothesized that ACPAs may directly target citrullinated C9 in RA, and possibly in ACPA+ FDR without RA. To test this, we citrullinated recombinant C9 with PAD and confirmed the presence of citrulline using a Rhodamine probe (phenylglyoxal, Fig. 3D, Figs. S6 and S7). Following this, we performed a western blot using high level ACPA+ RA sera as the primary antibody and observed an increase in reactivity to citrullinated C9 compared to the native form. We then measured anti-citrullinated C9 IgG in sera from RA, ACPA+ controls and ACPA- controls using ELISA and observed a heightened response in both RA and ACPA+ controls, suggesting that ACPA indeed targets citrullinated C9 (Fig. 3E).

**Fig. 4:**
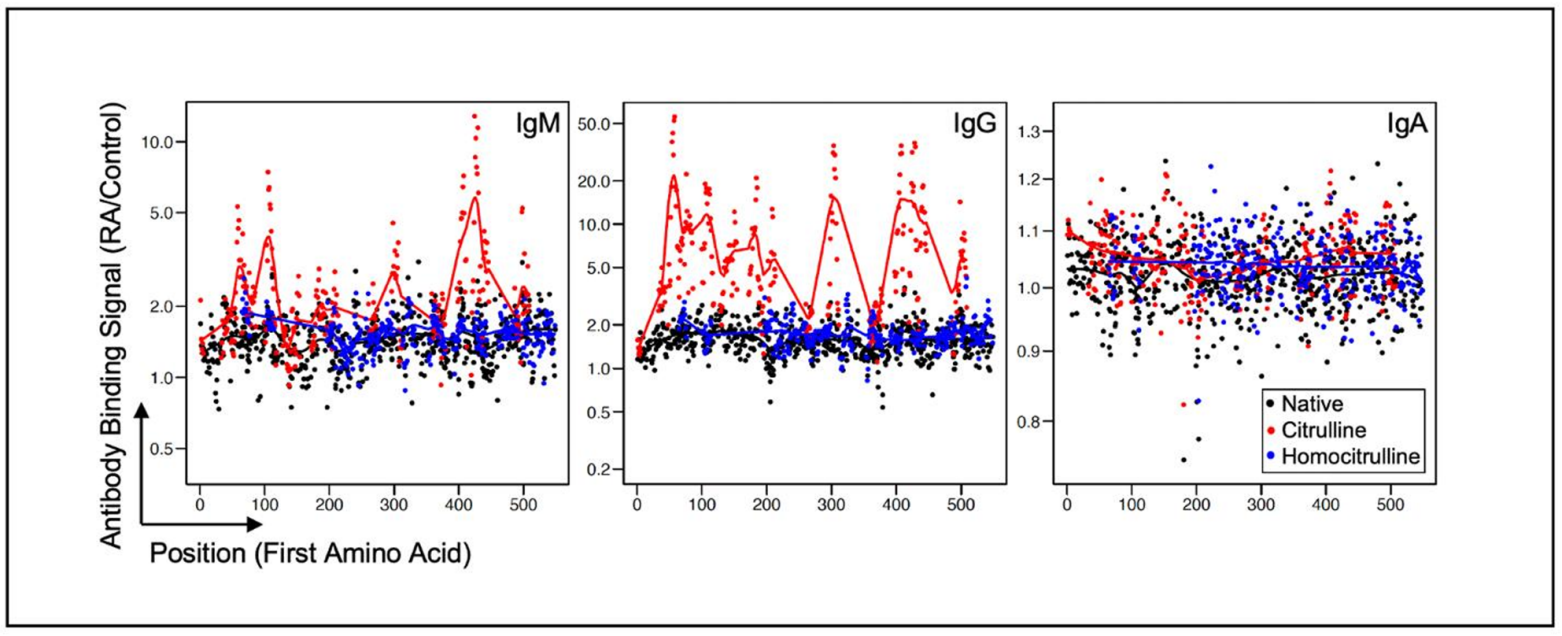
RA autoantibody responses to linear peptides of citrullinated and homocitrullinated C9. IgM, IgG, and IgA binding signal for anti-CCP+RF+ rheumatoid arthritis sera (*n* = 12) divided by age- and sex-matched control sera (*n* = 12) to all possible 12 amino acid linear peptides from C9 (Uniprot P02748) tiled on a peptide array. Correlations calculated using Pearson correlation. Differences in mean calculated with student’s t-test or ANOVA where appropriate

To evaluate antibody binding to linear epitopes of C9, which contains 33 arginine’s, we used antibody binding data from a previously published peptide array [10]. The array contained 12 amino acid linear peptides derived from C9 tiled at single amino acid increments. Peptides were in native form, a form in which arginines residues were replaced by citrullines, and a form in which lysines residues were replaced by homocitrullines, a known RA autoantigen that is structurally similar to citrulline [13]. Sera from seropositive RA and control participants were used to quantify IgM, IgG, and IgA binding to array peptides. We observed both IgM and IgG polyreactivity to multiple citrulline-containing C9-derived peptides, while IgA binding was minimal (Fig. 4, Table S2). We also observed that the binding to native and homocitrulline-containing C9-derived linear epitopes was limited. Overall, these data suggest that citrullinated C9 is a target for ACPA in RA and ACPA+ individuals without RA, but the upregulation of cit-C9 occurs exclusively in establish RA, possibly related to systemic inflammation.

### Citrullination may alter the structure of C9

**Fig. 5:**
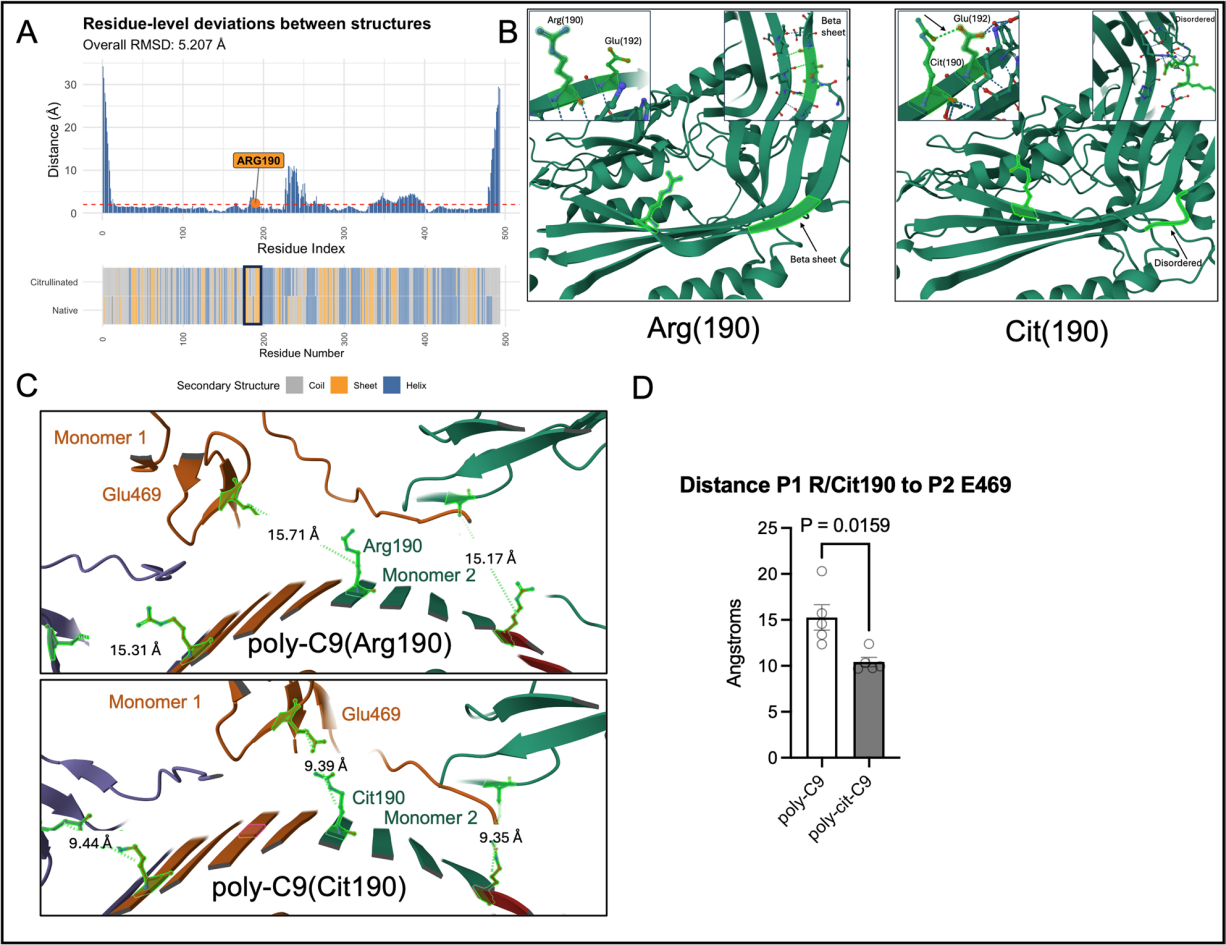
Citrullination of C9 alters the monomeric structure of C9. (**A**) Root mean-squared deviation (RMSD) along the citrullinated variant of the C9 monomer relative at site 190. The y-axis is amino-acid level differences in distance relative to the unmodified protein. (**B**) Unmodified (left) and citrullinated (right) monomers of C9 highlighting the interaction between Arg or Cit 190 and Glu (192) and loss of the Beta sheet in the structure. (**C**) Unmodified (top) and citrullinated (bottom) polymers (purple, orange, green) revealing a new interaction between Cit190 and Glu469 from an adjacent monomer and (**D**) which shows the calculated distance between these AA between polymer 1 and 2 in the unmodified and citrullinated polymers

Citrullination of proteins not only serves as a key step towards autoantigenicity in RA, but this PTM also influences the structure of proteins and protein-protein interactions. Complement C9 is the terminal product of the complement cascade which forms pores in the cell membrane of invading microbes [14]. This interaction occurs between multiple monomers (often between 10 and 12, called poly-C9) to form a single pore, which leads to osmotic lysis of cells through ion exchange. To determine pathologically relevant sites of citrullination in C9, we used a published proteomics RA synovial citrullinome dataset [15]. Here, a single citrullination was identified on the arginine at site 190 of C9. We undertook in-silico modeling using AlphaFold 3.0 of native and citrullinated C9 to investigate the structural changes mediated by citrullination. Superposition of citrullinated and native C9 produced an overall alpha Carbon root-mean squared distance of 5.207 Å, which was mostly localized to N- and C- terminal regions. However, additional deviations were observed near the citrullination site at 190 (Fig. 5A), as well as around Alanine (236) and Arginine (368). Visual inspection around the site of Arg190 revealed disruption of the β-sheet spanning residues Glu182 - Lys184, with per-residue deviations of 2.01 Å, 2.91 Å, and 3.72 Å, respectively, suggesting that citrullination at Arg190 induces the potential for structure perturbation in this region (Fig. 5B). Further, a new interaction between Glutamic Acid (192) and Citrulline (190) was observed, that was not apparent in the native structure. We also modelled poly-C9 (MAC), using the multimer of Arg190-C9 or Cit190-C9 and found that both forms of the protein successfully created a structure that resembled a pore (Fig. 5C). Citrullination resulted in some structural deviations (alpha-C RMSD 3.012 Å, Figure S9). The majority of the deviations were detected near site 190. Visual inspection of the multimeric structures revealed a new hydrogen bond formed between glutamic acid 469 and citrulline 190 (a bond not observed with arginine), drawing adjacent monomers closer together (Fig. 5D). Overall, these data suggest that citrullination of C9 in the synovial fluid in RA may impact the monomeric and multimeric structure of C9.

## Discussion

Citrullination is a ubiquitous PTM that plays a fundamental role in the functionality of multiple proteins and physiological processes. Citrullination is also a key process mediating the generation of pathological autoantigens in RA. It is not currently known why the generation of citrulline residues in specific proteins leads to pathogenic immune responses. We hypothesized that a broader understanding of the spectrum of circulating citrullinated proteins, particularly those that are overabundant in RA patients, may provide important insights into why specific citrullinated proteins become autoantigens and, in turn, how this may impact functionally on the networks in which these proteins participate. We used mass spectrometry paired with phenylglyoxal enrichment to better define the citrullinome in RA sera and compared it to the circulating citrullinome of unaffected individuals. Bioinformatics revealed distinct, highly correlated protein networks that were enriched in pathways suggestive of complement protein activation and host defense. In RA, the citrullinome was enriched in complement protein members. We also show that complement citrullination also leads to the generation of key autoantigens, specifically those targeting C9 of the terminal complex. Finally, we show that in-silico models of citrullinated and native C9 generate the pore forming membrane attack complex, but that these structures vary substantially in their quintenary structure.

The process of citrullination is mediated by a family of calcium dependant enzymes referred to as peptidyl arginine deaminases (PAD). These are calcium dependant enzymes of which there are several isoforms, yet PAD2 and PAD4 have garnered the most interest in their ability to generate autoantigens in RA specifically [16]. Mass spectrometry approaches have previously identified citrullinated protein in the RA synovium, many of which serve as key autoantigens in the disease [17]. Immunoprecipitation to pre-enrich for citrulline using RA synovial fluid also revealed an enrichment of citrullinated proteins and citrullinated sites [18]. There are however important limitations when identifying citrulline using mass spectrometry generated data, specifically distinguishing the small mass shift (0.98 Da) observed in citrullination and deamidation. The discovery of a phenylglyoxal probe [19] allows for highly confident profiling of citrullinated proteins in complex biospecimens. The first description of this approach revealed citrullination of Serpin (protease inhibitors) in RA tissue and synovial fluid, suggesting that serpin activity is greatly reduced by citrullination which may lead to uncontrolled destruction of joint structures/tissues [20].

We sought to build upon this approach by depleting highly abundant serum proteins which can enhance the identification of low expression proteins [21]. Using this method, we identified an over-abundance of citrullinated complement proteins in RA sera compared to controls. The complement cascade serves as a key component of the innate immune system and primarily through the formation of the terminal end-product called MAC (C5b-C9), a pore-forming structure which disrupts osmotic balance leading to cell lysis. In RA, complement activation appears to occur at sites of inflammation including the synovium [22], perhaps mediating joint damage [23], while others have shown that systemic complement protein synthesis is increased in RA [24]. ACPA itself may serve to activate the complement cascade through the classical and alternative pathways [25].

Antibodies targeting complement factor B [26], MBL [27], factor H [28], and citrullinated C1-inh have been described in RA patient sera [29]. We show that autoantibodies also target citrullinated C9, the major component of MAC. Interestingly, citrullination of C1-inh reduced its ability to inhibit specific proteins in the complement cascade, leading to dysregulated activation [29] which may fuel RA-related inflammation. Overall, this suggests a link between protein citrullination, activation of complement, and autoantibody formation in RA.

The development of anti-citrullinated protein antibodies (ACPA) is strongly associated with HLA-DRB1 shared epitope alleles, the major genetic risk factor for seropositive rheumatoid arthritis [30]. Although numerous citrullinated antigens have been described, commonly studied targets include histones, fibrinogen, vimentin and α-enolase. Importantly, ACPA fine specificities vary substantially between individuals with ACPA+ RA, and there is no evidence for a single dominant citrullinated autoantigen shared across all patients. Adding further complexity, other anti-modified protein antibodies, such as those recognizing homocitrulline (carbamylated proteins), are detected in subsets of both ACPA+ and ACPA- RA patients. Some ACPA exhibit promiscuous binding, characterized by relatively low affinity but broad reactivity to multiple citrullinated epitopes and, in some cases, homocitrullinated residues [31]. In contrast, other ACPA display high specificity, recognizing defined citrulline-containing motifs present in a limited number of proteins [32]. Notably, the local peptide context, including flanking amino acids, is a critical determinant of ACPA binding [33], supporting the concept that protein and site-specific citrullination events contribute to the development and diversification of autoimmunity in RA.

There are several important limitations to this study. As our focus was predominantly on the sera as a source of citrullinated proteins, we are unable to comment on the role of citrullinated proteins at other sites, such as the joint, lungs or intracellularly. Although using the phenylglyoxal-biotin approach provided a rich dataset of citrullinated proteins, we cannot be certain on the precise location of citrulline sites, as on-bead digestion with trypsin was used to generate peptides prior to MS analysis, leaving the labeled citrulline site attached to streptavidin. Hence, citrullinated proteins are inferred through the enrichment using the phenylglyoxal approach, and site specific citrullination data is not available, however the identification of site-specific citrullination will be the focus of future work. While confident identification of citrullinated proteins in complex specimens by mass spectrometry remains a challenge [34], recent advances are being made in this field [34, 35].

In summary, we show that the RA citrullinome is dominated by citrullinated complement proteins and that citrullinated C9 may serve as a key autoantigen in RA and individuals at-risk for RA (ACPA+ controls).

## Supplementary Information

Supplementary Material 1.

Supplementary Material 2.

Supplementary Material 3.

## Data Availability

No datasets were generated or analysed during the current study.
