# Supplementary material for: The rheumatoid arthritis citrullinome is enriched in antigenic complement proteins
Source: Arthritis Res Ther. 2026 Jan 28;28:56. doi: 10.1186/s13075-026-03750-9 (PMC12924597; doi:10.1186/s13075-026-03750-9)
Supplement: Supplementary file 1 — Supplementary Material 1. [file 13075_2026_3750_MOESM1_ESM.docx]

Supplemental Methods

*Mass spectrometry*

The samples were analyzed on an Orbitrap Exploris 480 instrument (Thermo Fisher Scientific, Bremen, Germany). The digests were introduced using an Easy-nLC 1200 system (Thermo Fisher Scientific) at 1 ug per injection. Mobile phase A was 0.1% (v/v) formic acid and mobile phase B was 0.1% (v/v) formic acid in 80% acetonitrile (LC-MS grade). Gradient separation of peptides was performed on a C18 [Luna C18(2), 3 μm particle size (Phenomenex, Torrance, CA)] column packed in-house in Pico-Frit (100 μm X 30 cm) capillaries (New Objective, Woburn, MA). Peptide separation was conducted using the following gradients: started with 2% of phase B, 2 – 6% over 5 minutes, 6 – 30% over 62 minutes, 30 – 45% over 7 minutes, 45 – 90% over 1 minute, with final elution of 90% B for 15 minutes at a flow rate of 300 nL/minute.

Data acquisition on the Orbitrap Exploris 480 instrument was configured for data-dependent method in a positive mode. Spray voltage was set to 2.6 kV, funnel RF level at 40, and heated capillary at 275°C. Survey scans covering the mass range of 380–1500 m/z were acquired at a resolution of 60,000 (at m/z 200), with a normalized automatic gain control (AGC) target of 300% and an auto maximum ion injection time. This was followed by MS2 acquisition at a resolution of 15,000 with an intensity threshold kept at 2e4. During MS2 acquisition, the 20 most abundant ions were selected for fragmentation at 30% normalized collision energy. AGC target value for fragment spectra was set to standard with an auto maximum ion injection time and an isolation width set at 1.6 m/z. Dynamic exclusion of previously selected masses was enabled for 20 seconds, charge state filtering was limited to 2–6, peptide match was set to preferred, and isotope exclusion was on.

*Citrullinated complement proteins*

To measure the relative abundance of citrullinated complement proteins in serum an in-house ELISA was used. The capture antibody (mouse anti-citrullinated protein antibody, SMC-501D, 1:1000) was coated overnight at 4^o^C on a 96-well high binding plate. Following a wash step, the plate was blocked with 1% Bovine Serum Albumin (BSA), serum (1:100 dilution in 1% BSA) was added to each well and incubated overnight at 4^o^C with gentle rocking. The plate was then washed 3 times with PBS-T and a detection antibody was added (rabbit anti-CFI, PA5-75509 or rabbit anti-C9, PA5-29093) for 60 minutes at RT with gentle rocking. The plate was then washed 3 times with PBS-T and the secondary antibody (goat anti-rabbit HRP, 1:10000) was added for 60 minutes at RT with gentle rocking. Finally, the plate was washed (5x), developed using Tetramethylbenzidine and stopped with 2M Hydrochloric Acid and subsequently read by *absorbance* (450 nm) using a Synergy H6.

*Complement protein Immune complexes and antibodies to citrullinated C9*

To measure immune complexes to complement proteins in serum an in-house ELISA was used. 96-well plates were coated with a capture antibody (1:1000 rabbit anti-complement factor 1 or rabbit anti-complement 9) overnight at 4^o^C. After blocking with 1% BSA for 60 minutes, and sera (1:100) was added overnight at 4^o^C with gentle rocking. After 3 washes, a secondary antibody (goat anti-human IgG, 1:10000) was added and incubated at RT for 60 minutes. Following 5 washes, the plate was then developed using Tetramethylbenzidine and stopped with 2M Hydrochloric Acid and read by *absorbance* (450 nm). To citrullinate complement protein C9, recombinant C9 (abcam) was exposed to PAD2 (50 U, sigma) at 37^o^C for 60 minutes. Confirmation of citrullination was visualized using a rhodamine phenylglyoxal probe using methods as described in prior publications and visualized by in-gel fluorescence^11^. Similarly, C9 and citrullinated-C9 were run in a separate gel (Sodium dodecyl sulfate - polyacrylamide gel electrophoresis) and transferred to a nitrocellulose membrane for analyze by Western Blot using serum from an RA patient with high levels of anti-citrullinated protein antibody to confirm reactivity (secondary ab goat anti-human IgG HRP).

Total C9 was immunoprecipitated from pools of serum from RA (n=3), FDR (n=3) and SLE (n=3) using rabbit anti-complement 9. In brief, anti-C9 IgG were incubated with Protein A beads overnight at 4oC with gentle rocking. The beads were washed, then incubated with pools of sera overnight at 4oC. Following incubation, the beads were washed with 2 volumes of columns of 0.1 M Sodium Acetate buffer 0.2M NaCl 3 times. After washing, proteins bound to the beads were eluted using an IgG elution buffer pH 2.7 (Thermo Scientific cat# 21004) and following neutralization, a western blot was performed using serum from an RA patient with high level ACPA (1:5000).

Subsequently, citrullinated C9 was coated on a 96-well high binding plate (10 ng per well) overnight at 4^o^C. Serum (1:100) was then added after 60 min of blocking with 1% Bovine Serum Albumin and incubated overnight at 4^o^C with gentle rocking. After 3 washes, a secondary antibody (goat anti-human IgG, 1:10000) was added and incubated at RT for 60 minutes. Following 5 washes, the plate was then develop using TMB and stopped with 2M HCl and read by absorbance (450 nm).


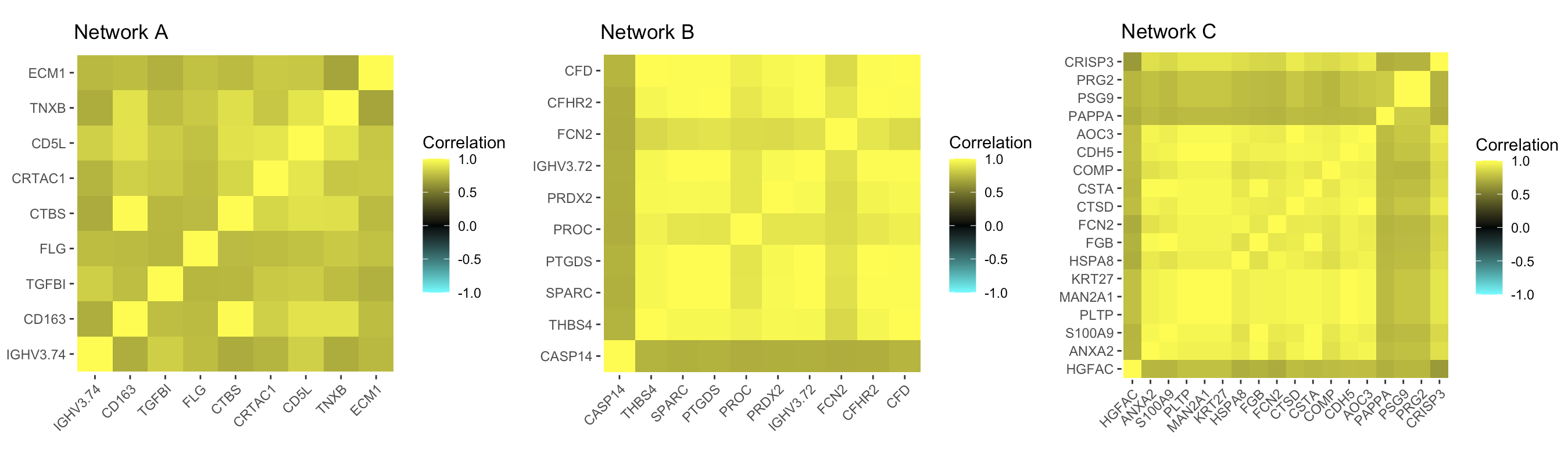


Figure S1: Heatmap of each highly correlated Network (A, B, C) of citrullinated proteins derived from serum samples using mass spectrometry. Data were generated using igraph in R.


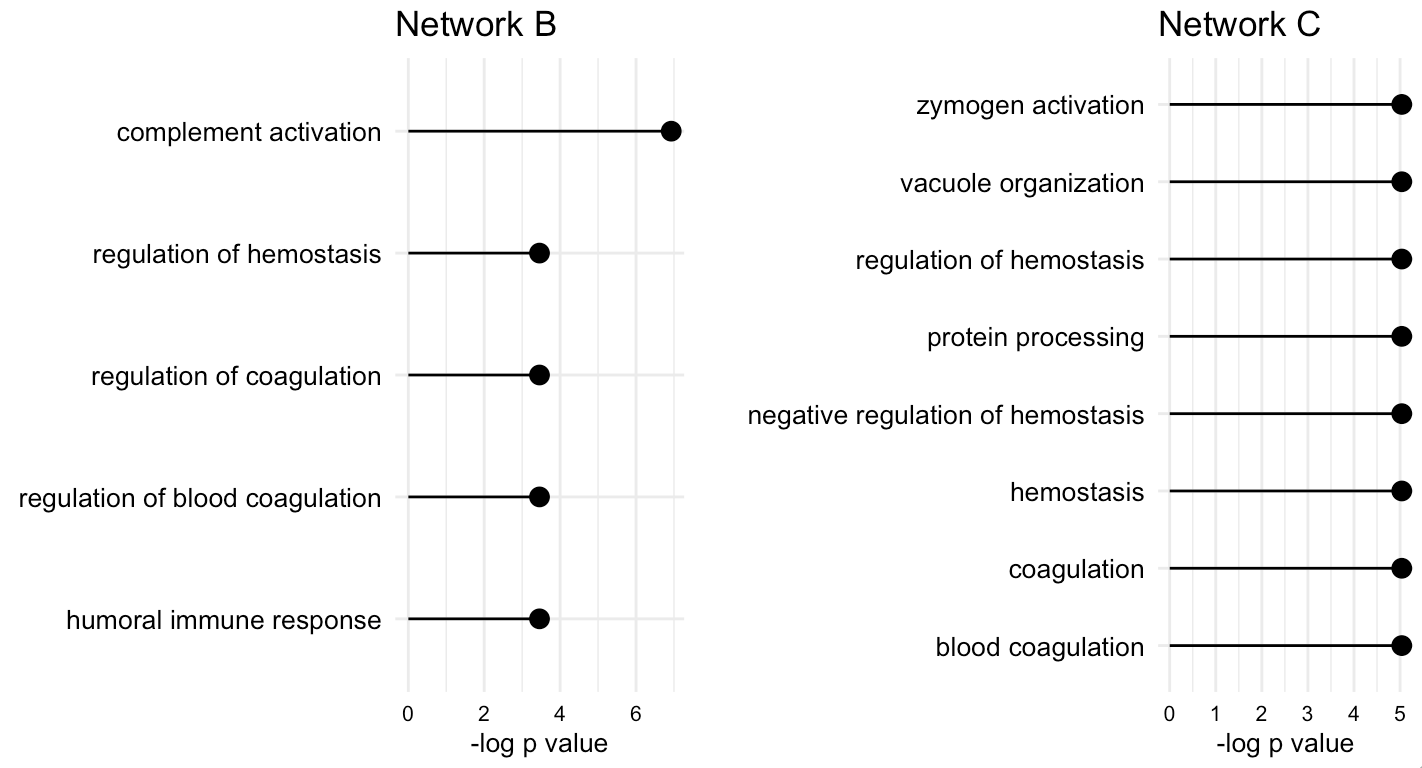


Figure S2: Gene ontology enrichment in Network B and C. Network A was not enriched in any pathways that achieved statistical significance.

Figure S3: Correlation coefficients of Network A, B and C associated citrullinated proteins as a measure of co-expression in FDR and RA patients. Differences measured using Wilcoxon-rank sum test.

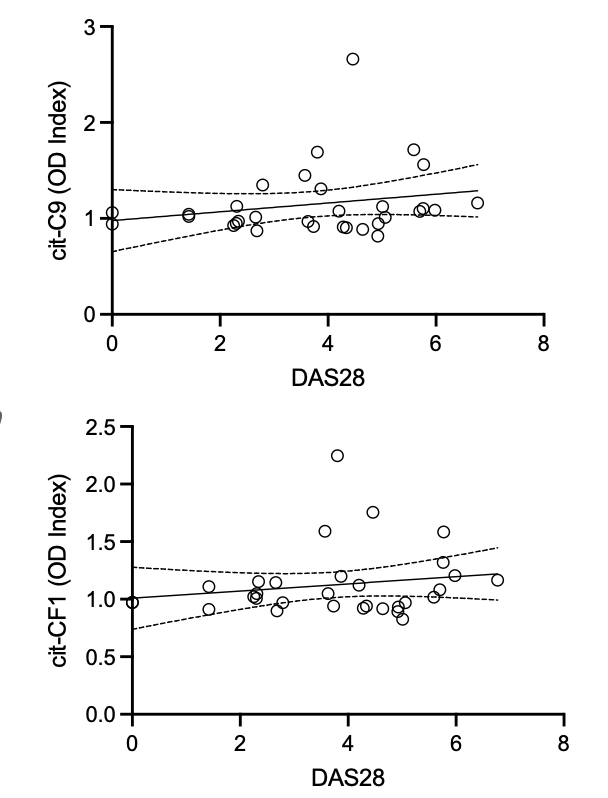


Figure S4: Spearman correlation between serum cit-C9 and cit-CF1 (ELISA) and DAS28 scores. DAS: Disease activity scores.


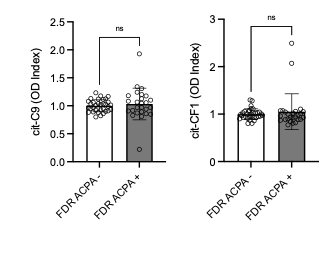


Figure S5: Levels of serum cit-C9 and cit-CF1 in ACPA+ FDR measured by ELISA. Differences determined using Wilcoxon-Rank sum test.


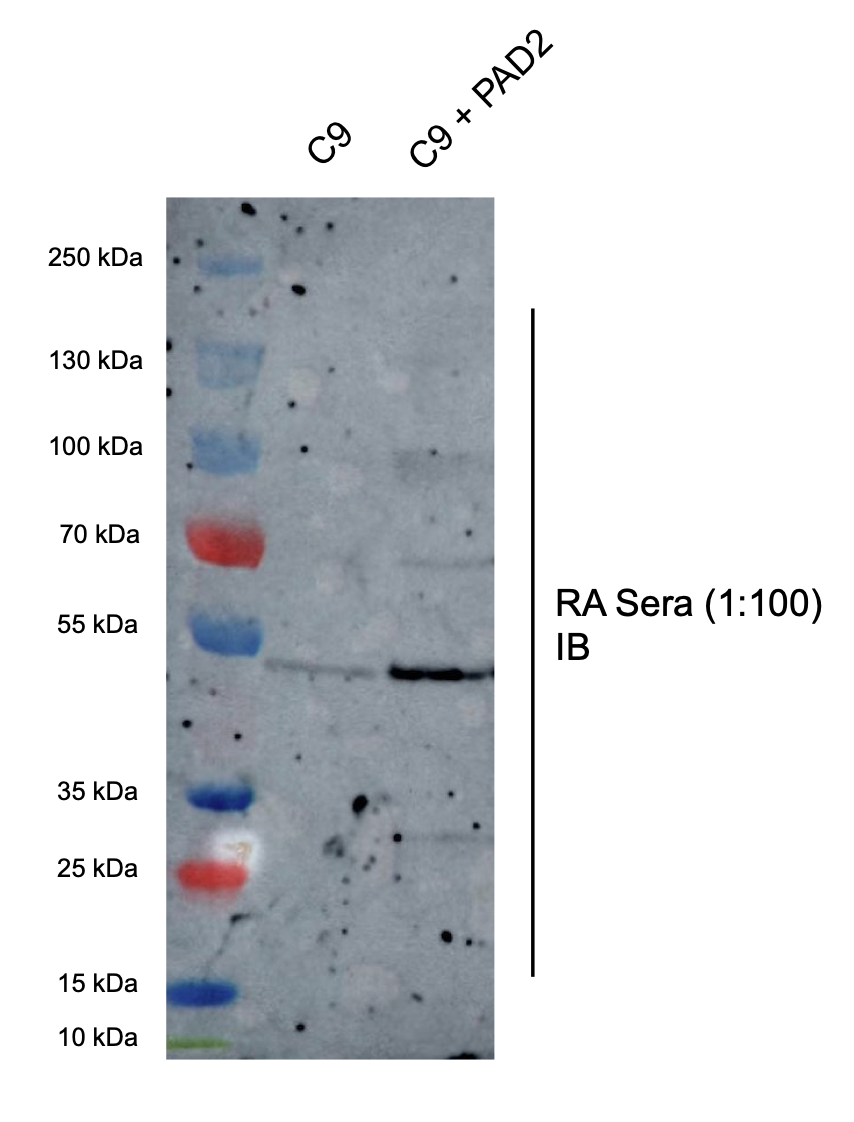


Figure S6: Uncropped blot of western blot with RA sera and C9 or citrullinated C9.


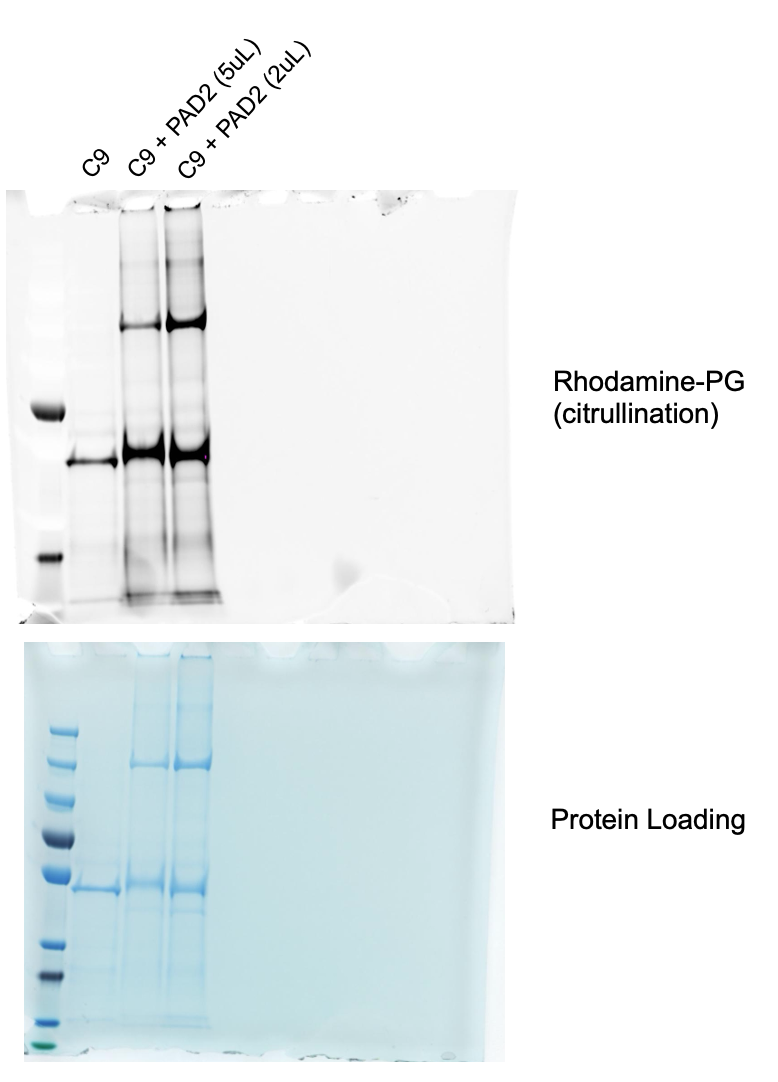


Figure S7: Uncropped blot of in-gel fluorsence using a citrulline specific rhodamine probe (PG) and protein loading by Sypro Ruby.


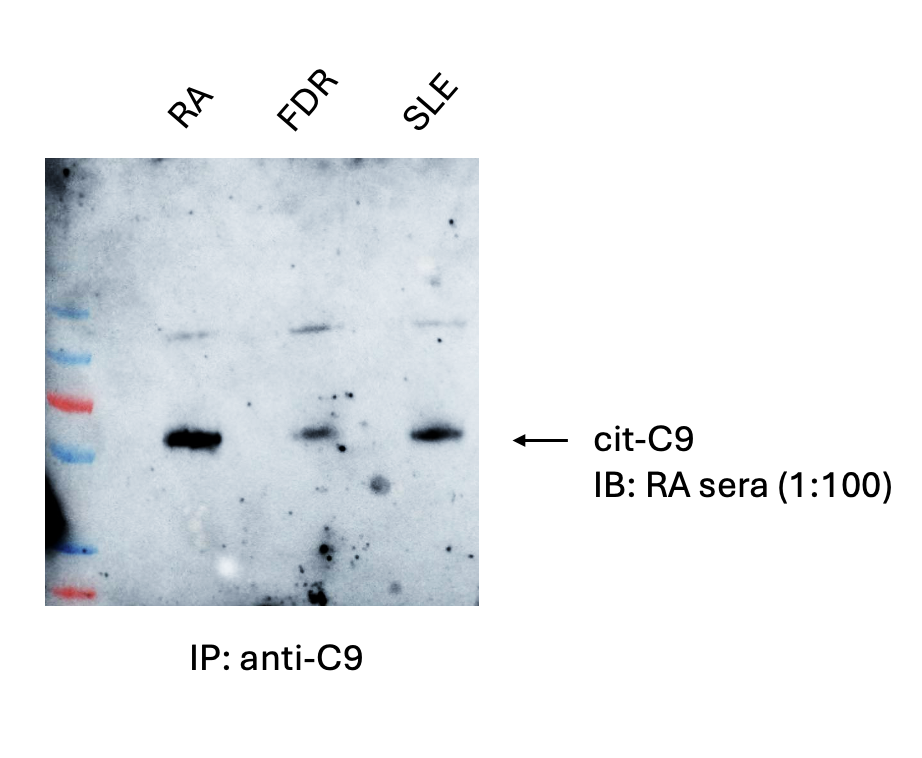


Figure S8: Uncropped blot of immunoprecipitated C9 from pooled sera (n=3) probed by RA sera from an ACPA+ patient (1:100).


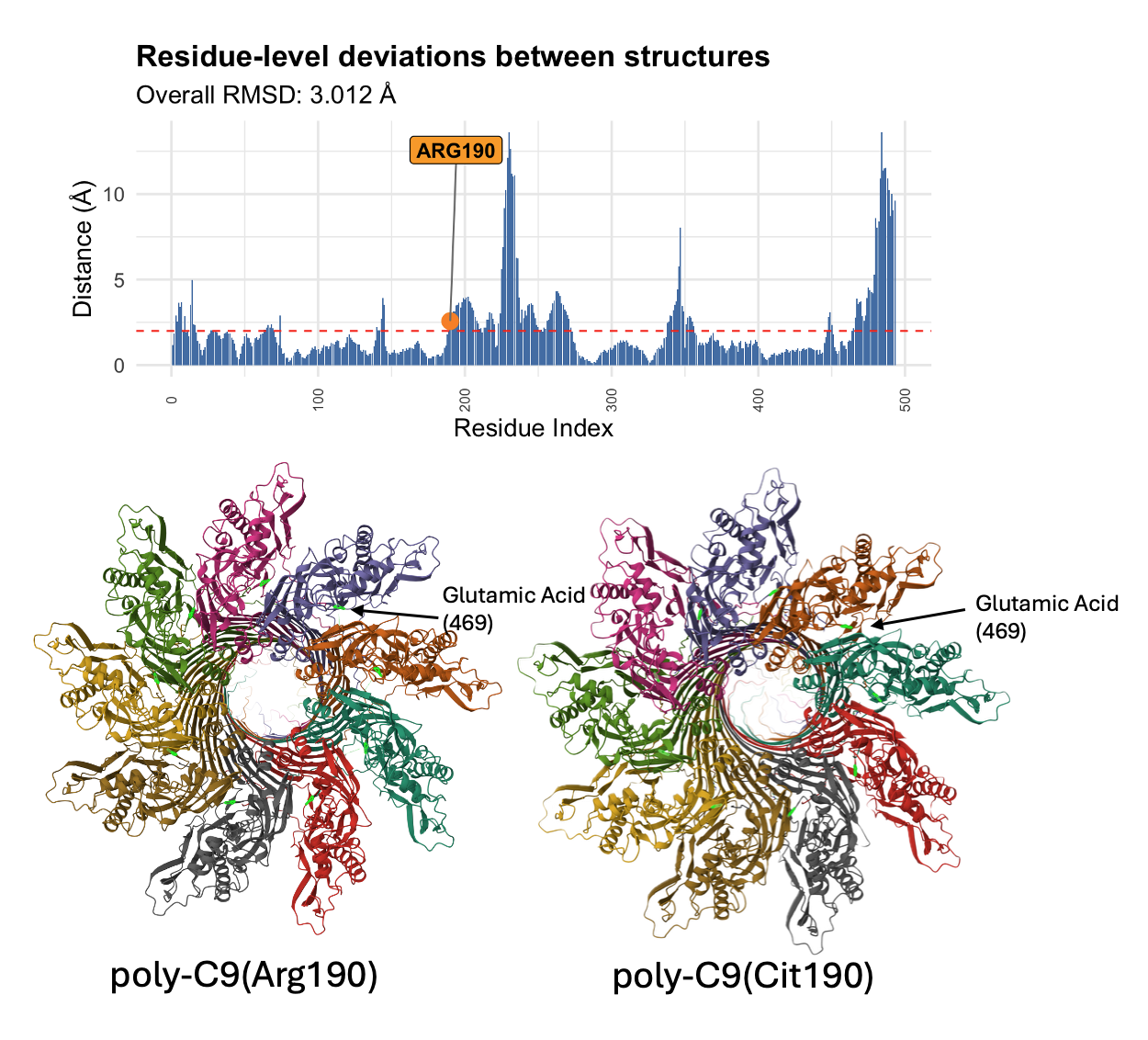


Figure S9: Residual-level deviation between aligned polymers of C9 and cit-C9 (9 monomers) based on alpha 3.0 folding. Visualization were generated using PDB viewer. Each colour represents a distinct monomer.
